# Barriers and facilitators to equitable implementation of long-acting ART for adolescents and youth with HIV in low- and middle-income settings

**DOI:** 10.11604/pamj.2024.49.53.45322

**Published:** 2024-10-23

**Authors:** Nadia Adjoa Sam-Agudu, Chibueze Adirieje, Allison Lorna Agwu, Natella Rakhmanina

**Affiliations:** 1 International Research Center of Excellence, Institute of Human Virology Nigeria, Abuja, Nigeria,; 2 Department of Paediatrics and Child Health, School of Medical Sciences, University of Cape Coast, Cape Coast, Ghana,; 3 Global Pediatrics Program and Division of Infectious Diseases, Department of Pediatrics, University of Minnesota Medical School, Minneapolis, USA,; 4 Division of Infectious Diseases, Department of Pediatrics, Johns Hopkins University School of Medicine, Baltimore, USA,; 5 Division of Infectious Diseases, Department of Medicine, Johns Hopkins University School of Medicine, Baltimore, USA,; 6 Division of Infectious Diseases, Children's National Hospital, Washington DC, USA,; 7 School of Medicine and Health Sciences, The George Washington University, Washington DC, USA,; 8 Elizabeth Glaser Pediatric AIDS Foundation, Washington DC, USA

**Keywords:** Adolescent, HIV, adherence, antiretroviral therapy, low-and middle-income countries, implementation science, Africa

## Abstract

Recent approvals of long-acting (LA) antiretroviral treatment (ART) support an innovative alternative to daily oral pills that can improve adherence and treatment outcomes among adolescents and youth (AY) with HIV. We solicited stakeholder feedback on the implementation of LA ART for AY in low-and middle-income countries (LMICs) through a consensus-building forum at the 2022 International Workshop on HIV and Adolescence. We used the nominal group technique to generate, record, discuss, vote on, and rank perceived barriers and facilitators to implementing LA ART for AY. All in-person attendees were invited to participate and were assigned to six groups, each representing an intentional mix of AY, clinicians, researchers, program implementers, and policymakers. We collected self-reported de-identified demographics and group rankings of barriers and facilitators. Responses were coded and categorized using the social-ecological model’s five levels of influence. One hundred and thirty-seven (137) Workshop delegates (67.9% male, 27.7% female; 0.7% non-binary, and 46.7% less than 35 years old) participated in the group discussions. A large proportion of participants (51.9%) reported working in public health/program implementation. Most participants (88.4%) were from and/or worked in the African region. We identified 55 barriers and 48 facilitators of LA ART implementation and ranked them in social-ecological categories of public policy, community, institutional/organizational, interpersonal, and individual levels. The highest number of ranked barriers was at the institutional/organizational level. The themes of “equitable access” and “choices of ART” were cross-cutting across individual and interpersonal levels. Other cross-cutting themes were the “cost of LA ART” and the ”need for funding and sustainability of LA ART programs”. Proposed facilitators addressed identified barriers at each social-ecological level of influence and emphasized peer engagement. Our nominal groups identified key barriers and proposed facilitators at five different social-ecological levels, which can inform implementation science-guided design and equitable implementation of youth-centered LA ART in LMICs and globally.

## Introduction

The last decade has seen an improvement in the trend of adolescent AIDS-related mortality [1]. However, there continues to be a disproportionately high rate of adolescent AIDS-related deaths in high-burden African countries (9.92 per 100,000) where ~80% of adolescents with HIV reside, vs globally (2.3 per 100,000) [2]. This disproportionately high HIV-related morbidity and mortality has been ascribed to inadequate access to treatment, low treatment adherence, and health services poorly responsive to adolescent needs in Africa [3]. Adolescents and youth (AY) with HIV face many challenges in adherence to daily oral antiretroviral treatment (ART) and have some of the worst treatment outcomes compared to other age groups of people with HIV [4,5]. To achieve the UNAIDS 95-95-95 goals, adolescents need access to a variety of ART modalities in addition to adherence and retention interventions delivered via innovative, adolescent-responsive strategies [6-9].

Recently approved long-acting (LA) ART for HIV treatment in adolescents and adults represents an attractive alternative to daily oral pills in HIV treatment. These approvals open up opportunities for an innovative biomedical intervention to improve ART adherence and treatment outcomes among AY in low- and middle-income countries (LMICs) [10]. Current options for LA ART comprise several therapeutic choices: a full regimen combination of an integrase strand transfer inhibitor cabotegravir (CAB) and a non-nucleoside reverse transcriptase inhibitor rilpivirine (RPV) (administered as two intramuscular injections every one to two months with optional oral lead-in) [11], monoclonal antibody ibalizumab-uiyk (administered as an intravenous infusion or push every two weeks) [12], and a capsid inhibitor lenacapavir (administered as a subcutaneous injection every six months) [13] to be used in combination with other antiretroviral drugs. A United States study of 303 AY aged 13-24 years showed high AY interest in LA ART, with the highest interest among those with elevated viral loads [14]. Based on interim data from the IMPAACT MOCHA adolescent study (ClinicalTrials.gov #NCT03497676), LA CAB/RPV ART was approved by the United States (US) Food and Drug Administration in March 2022 and has since been in use among virologically suppressed adolescents and adults ≥12 years old weighing ≥35 kg and without CAB/RPV drug resistance [11]. Additional data on safety, acceptability, tolerability, and pharmacokinetics are anticipated for adolescents 12-17 years from MOCHA in the US, South Africa, Botswana, Puerto Rico, Uganda, and Thailand. Phase III studies among adults with HIV in the USA, Spain, Australia, and seven high-burden African countries demonstrated sustained viral suppression and high acceptance of LA CAB/RPV ART [15-19].

Given its injectable mode of administration and favorable dosing schedule, LA CAB/RPV could become an important option for sustained ART among AY in LMICs [20-25]. Early implementation experience in high-income countries [26-29] can inform some of the programming in LMICs, however, timely stakeholder engagement in LMICs is crucially important to achieve global success in introducing and scaling up this new treatment modality, especially among AY.

A few studies emerging from LMICs are evaluating preferences for, and acceptability of future LA ART among adolescents and youth [30], heralding the timeliness of engaging adolescents and other stakeholders in decision-making regarding this new treatment modality. We aimed to elicit facilitators and barriers to the implementation of LA ART for adolescents and young people with HIV in LMIC settings through a consensus-building forum conducted at the 2022 International Workshop on HIV and Adolescence.

## Methods

**Figure 1:**
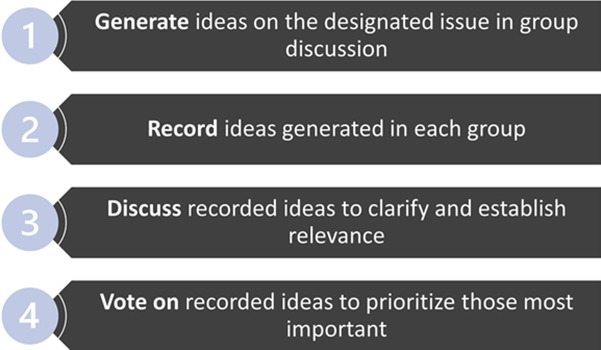
process of the nominal group technique for reaching consensus among stakeholders

This consensus-building forum was conducted during the in-person International Workshop on HIV and Adolescence held in Cape Town, South Africa on 5^th^ and 6^th^ October 2022 [31]. To address our objective, we adopted the nominal group technique, a structured small group discussion that aims to reach a consensus on an issue among stakeholders [32,33]. This technique combines both qualitative and quantitative methods and is designed to minimize domination of the discussion by individual participants, for example, those with more social, economic, or academic power. The nominal group process has four main steps: generate ideas, record the ideas, discuss the ideas, and vote on the ideas to rank them [32] (Figure 1). A moderator poses a question or questions to participants, who are then asked to prioritize responses given by all group members using numerical rankings. The group comes to a consensus on the ranked responses before the responses are finalized. All rapporteurs, selected by the groups, are then invited to present a summary of their group´s final consensus on the discussion questions to all workshop attendees in a plenary session. The nominal group discussions were held during a 60-minute session on day 2 of the workshop.

**Workshop delegate recruitment:** a total of 263 registered delegates attended the Workshop, all of whom were invited to participate in the forum. Before the nominal group discussions, there were three brief presentations totaling 20 minutes: the first presentation by author AA reviewed experiences and data from the US on adolescent preferences for LA ART; NR described practical experiences in administering LA ART to adolescents and young adults with HIV; and NASA explained the nominal group process to all attendees, informing them of the intent to write a report and requesting their voluntary participation. Workshop delegates who proceeded to participate in the group discussion after this point were considered to have provided consent. After these three presentations, an opportunity was given for attendees to ask any clarifying questions. Of note, there were two plenary day 1 presentations on HIV prevention and treatment for AY which included information on LA antiretrovirals [31].

**Data collection:** workshop delegates were assigned to six groups, each representing an intentionally diverse mix of youth, clinicians, researchers, program implementers, and policymakers. Group assignments were based on participants´ self-identification of their role or job description during Workshop registration. Two moderators were assigned per group: a youth implementer or advocate and a senior researcher, implementer, or clinician with expertise in adolescent HIV. Each group nominated a rapporteur to document group member responses and rankings for questions posed by the moderators. Group discussions were conducted for approximately 40 minutes. A standard form was used to collect self-reported de-identified demographic data from each participant, including region and country of work and/or residence, gender, age range, and area of focus for their work. Participants were instructed (verbally and in writing) not to write any identifying information on their forms. Moderators used a second, separate standard form to document group responses and rankings related to the study questions. There were no pictures taken, nor video or audio recordings; all data collected were exclusively hand-written on standard hard-copy forms and subsequently transferred to an Excel sheet for analysis. The key questions posed to participants for group discussion were: i) what are the barriers to implementing (rolling out) long-acting ART for adolescents and young adults in your country or region? Write down all barriers, then rank the top three; ii) what are the facilitators, or potential solutions in addressing gaps to implement (roll-out) long-acting ART for adolescents and young adults in your country or region? Write down all the facilitators/potential solutions, then rank the top three.

**Data analysis:** demographic data were analyzed by descriptive statistics. Nominal group technique responses and rankings regarding barriers and facilitators to the implementation of LA ART were coded and categorized using the social-ecological model. This model considers the different levels of influence on the adoption, uptake, or implementation of health behavior and/or intervention [34]. The five levels of influence applied to this analysis were: individual (regarding the adolescent with HIV); interpersonal (family, friends, and social networks of the adolescent); institutional/organizational (health facilities, schools, religious centers, workplaces); community (culture, informal or formal social norms, and community as a place of residence); and health-related policy (national, state, and other local laws). Group responses were also coded and categorized according to cross-cutting issues at two or more of the social-ecological levels. The four-member analysis team (NASA, CA, ALA, and NR) coded and categorized the nominal group technique data independently; individual analyses were then merged and discussed as a team. Any conflicting or outlying areas of analysis were voted on for consensus.

**Ethical considerations:** ethical approval was not sought for the study, given that participants were discussing questions posed at a public forum during a scientific workshop and that demographic data were voluntarily self-reported without any personal identifiers. The findings of this forum are being disseminated as a conference report.

## Results

A total of 137 Workshop delegates (52.1% of the 263 in-person attendees) participated in the nominal group discussions, of whom 93 (67.9%) self-reported as male, 38 (27.7%) as female, and 1 (0.7%) as non-binary (Table 1). Concerning age, 11 (8.0%) participants reported being 20-24 years old, 53 (38.7%) were 25-34 years old, and 68 (49.6%) were ≥35 years old; there were no participants <20 years old. In terms of profession or roles, the largest proportion of participants (49.6%) reported working in public health or program implementation.

**Table 1:** demographic profile of participants in nominal groups, N=137

Characteristic	n	%
**Gender**		
Male	93	67.9
Female	38	27.7
Transgender/non-binary	1	0.7
Undisclosed	5	3.6
**Age group (years)**		
10-19	0	0.0
20-24	11	8.0
25-34	53	38.7
≥35	68	49.6
Undisclosed	5	3.6
**Profession/practice**		
Public health/program implementation^a^	68	49.6
Research^a^	49	35.8
Public health/program implementation and research	6	4.4
Public health/program implementation and policy	2	1.5
Research and policy	1	0.7
Policy only	3	2.2
Activist^b^	11	8.0
Other^c^	7	5.1
Undisclosed	6	4.4

a Includes all participants who indicated this exclusively or in combination with another occupation (may add up to >100%); ^b^includes 1 participant engaged in both activism and research; ^c^includes advocacy (1), clinician (1), clinical psychologist (1), industry (2), pharmaceutical (1), and marketing (1)

**Figure 2:**
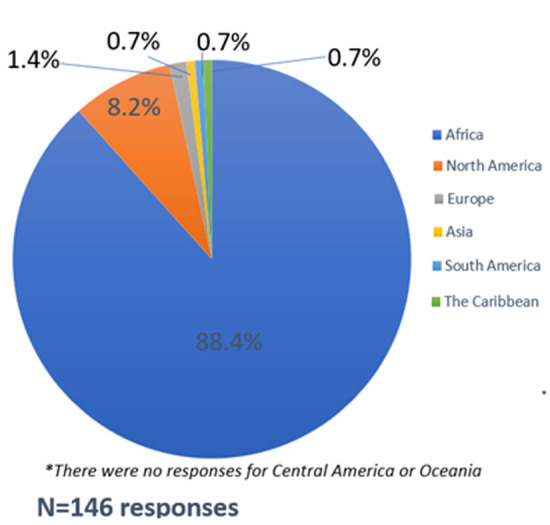
region of primary residence and/or work among participants based on responses

**Figure 3:**
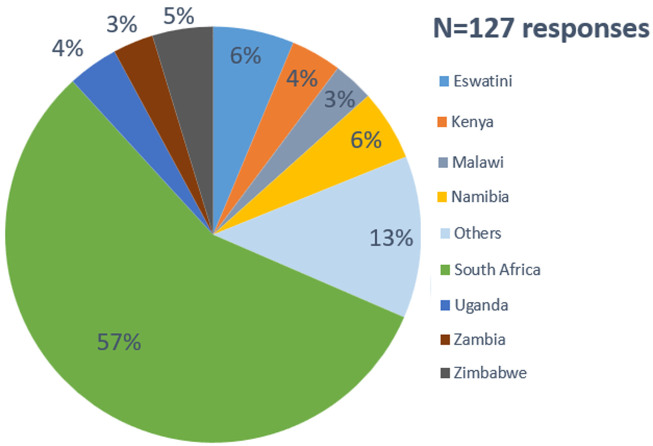
country of primary residence and/or work among participants living and/or working in Africa; “other” refers to countries that each represent 1-2% of responses provided (Botswana, Burundi, Cameroon, DR Congo, Ethiopia, Mozambique, Rwanda, Senegal, Somalia, and Tanzania)

In terms of region of primary residence and/or work, most participants (88.4%) were from, and/or worked in the African region, followed by North America (8.2%) and Europe (1.4%) (Figure 2). Four percent of all participants reported living and working in both Africa and North America. The pie chart shows regional representation at the workshop; some participants lived or worked in more than one region, with each recorded separately. There were no participants from Oceania. At the country level, South Africa had the most representation (57%) among all participants, which is expected given that the conference was held in Cape Town. Eswatini (6.3%), Namibia (5.5%), Zimbabwe (4.7%), Kenya (3.9%), and Uganda (3.9%) rounded up the top six African countries by participant representation (Figure 3). Among the six non-African countries reported, the USA was represented by nine (60%) of 15 participants, two for the United Kingdom (13.3%), and one each (6.7%) for Haiti, Malaysia, France, and Switzerland (not displayed).

**Figure 4:**
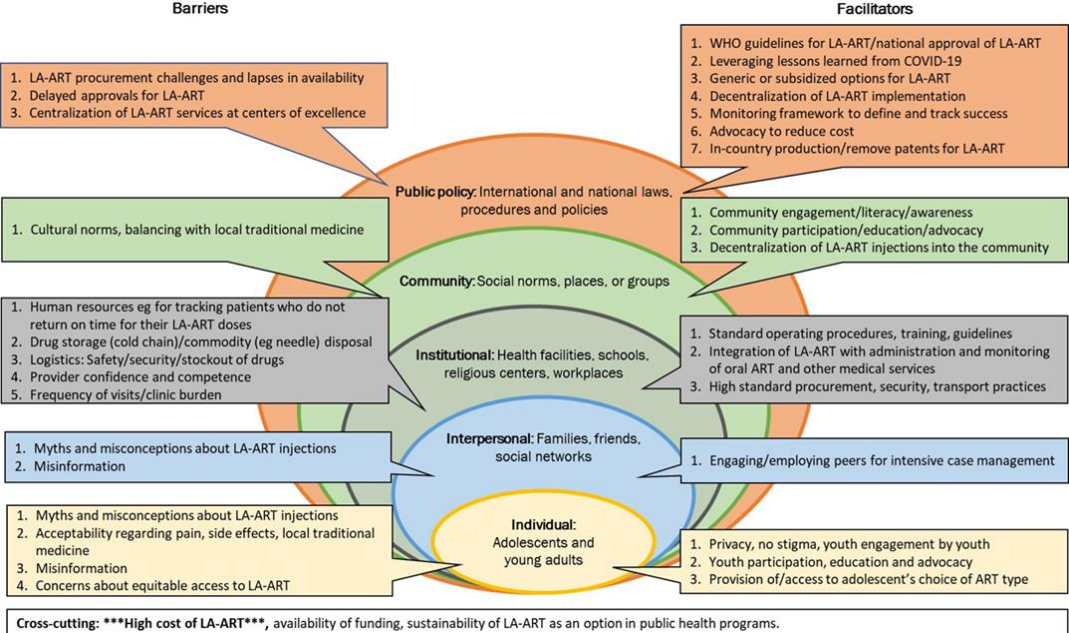
top-ranked barriers and facilitators to implementing long-acting (LA) antiretroviral treatment (ART) for adolescents and youth

**Consensus on long-acting antiretroviral treatment for adolescents and youth:** a total of 55 barriers and 48 facilitators relevant to LA ART implementation were provided by the groups. The data were collated and then merged to harmonize similar concepts. All barriers and facilitators were then sorted according to the five levels of the social-ecological model, in addition to a cross-cutting category. The top three ranked barriers and facilitators from each group were extracted, collated, and sorted according to the social-ecological model (Figure 4). These collated factors were listed in order of priority, according to the number of times they were listed in individual group rankings across the six groups. There are less or more than three barrier or facilitator factors reported at some social-ecological levels because the number of collated factors ranked at these levels was either relatively low or relatively high across the six groups. To minimize analytical bias, social-ecological levels were not specified during data collection; these levels were determined only during analysis. “Cost of LA ART/procurement challenges” was listed in the top three barriers for all six groups.

The highest-ranked public policy barriers included delayed approval and challenges with procurement, availability, and centralized distribution of LA ART at national levels. To mitigate these barriers, the groups prioritized several strategies including clear national and World Health Organization (WHO) recommendations on the use of LA ART, patent-free in-country production, use of generic or subsidized reduced-cost CAB/RPV, and decentralization of LA ART procurement and delivery services. Further supportive public policy strategies proposed included close monitoring and evaluation of treatment outcomes and applying lessons learned from testing and immunization campaigns in the COVID-19 pandemic response. Prominent barriers at the community level included negative cultural norms and beliefs around injections and the use/promotion of local traditional medicines instead of ART for HIV treatment. To address this challenge, groups highlighted the need for early community awareness and engagement, education and support, participation and advocacy, and importantly, decentralization of LA ART within the community.

The highest number of ranked barriers was at the institutional/organizational level (Figure 4) and included a lack of confident and competent personnel to administer injections and track and support AY on ART. In addition, the complex logistics of drug storage and delivery and the expected increased clinic visit burden were cited as barriers affecting healthcare providers and facilities. The proposed solutions included periodic provider training, development of standard operating procedures, integration of LA ART within oral ART programs and other medical services, and optimized secured procurement and transportation of the LA drugs.

Misinformation, misconceptions, and myths about LA ART were prominent barriers at the interpersonal and individual levels. Participants emphasized youth participation and advocacy, and engaging peer advocates for service delivery using intensive case management models. The themes of “equitable access” and “choices of ART” were cross-cutting at the individual and interpersonal levels for all six groups. Other cross-cutting themes were the high cost of LA ART, and the need for funding and sustainability of LA ART programs once established.

## Discussion

We report consensus findings on the implementation of LA ART among adolescents and youth with HIV from a large group of stakeholders (nearly 50% less than 35 years old) attending an international workshop on HIV in young people. A major strength of this report is that participants included youth advocates and multidisciplinary frontline programmatic and research staff working with youth across multiple LMICs, including disproportionately affected countries in Africa. Our nominal group technique and analysis identified key barriers at five different social-ecological levels. We synthesized these barriers and the proposed tangible solutions and strategies for consideration when designing youth-centered LA ART programs, especially those relevant to African countries.

Along the five social-ecological levels, several recurrent themes emerged relating to barriers and facilitators. Not surprisingly and following published multi-country analysis on considerations for the rollout of novel ART [35], the highest number of ranked barriers in our analysis was at the institutional/organizational level. These barriers included the need for a competent workforce and confident healthcare workers, an increased number of patients at clinic visits, changes in clinic flow and patient follow-up protocols, and multiple logistics of cold-chain storage of LA CAB/RPV and disposal of supplies. These barriers align and resonate with the body of evidence on the ongoing need for multi-level health system strengthening and investment in human resources [36]. Based on participants´ feedback and suggested pathways for LA ART implementation, we argue that integration of LA ART into existing ART programs provides a unique opportunity to update and optimize HIV-related provider training, standard operating procedures, and patient outcomes monitoring, and to intensify focus on the quality of clinical care [36,37].

Reminiscent of recent experiences with COVID-19 vaccines [38,39], misinformation and misconceptions about LA ART within communities and among AY were perceived as prominent barriers at interpersonal and individual levels. Lack of attention to interpersonal and social dynamics limits our understanding of the effective strategies needed to expand and scale up ART access, for example in high HIV burden African countries [40]. As suggested by Myburgh *et al*. it is not enough to place a predominant focus on health system infrastructure when expanding ART access; innovative individual and interpersonal-level strategies are additionally required to “increase the number of clients on ART while sustaining current processes to retain all in care” [40]. Based on the individual and interpersonal level solutions provided by our participants, providing transparent and accurate information, addressing myths and misperceptions, optimizing peer education, engagement, and advocacy, and reducing/eliminating stigma will all be crucially important for a successful LA ART rollout for AY. These barrier-focused solutions are consistent with the principles of differentiated service delivery and high-quality care in HIV programs as described by the WHO [37,41].

The public policy barriers and solutions described highlight the need for clear global and national guidance on the use of LA ART and facilitated access to patent-free generic or subsidized LA CAB/RPV formulations through decentralized mechanisms. These solutions depend on the affordability of LA ART formulations and equitable access in LMICs, similar to what has been negotiated and achieved for LA CAB pre-exposure prophylaxis (PrEP) [42,43]. As with LA CAB PrEP [44], cost should not be allowed to derail the scale-up of new ART modalities and their availability to eligible patients, including AY, for whom they hold promise to improve adherence and treatment outcomes [21].

Our consensus findings provide valuable information for the implementation science-guided roll-out of LA ART for AY in LMICs. This can be facilitated with technical support from local implementation science consortia such as those of the NIH/Fogarty Adolescent HIV Implementation Science Alliance (AHISA). Participants identified and ranked barriers and facilitators across individual, interpersonal, institutional, community, and public policy domains. The results and analysis are extremely useful for identifying, selecting, and developing highly relevant implementation strategies to minimize barriers and enhance facilitators across multiple social-ecological levels. In addressing the determinants (barriers and facilitators) of successful implementation, implementation strategies facilitate desired implementation outcomes. For AY-targeted LA ART, these outcomes would include acceptability among AY and other key stakeholders, uptake among AY, adoption by healthcare systems and providers, feasibility, fidelity, costs, and sustainment/sustainability [45,46].

**Limitations:** our report is not without limitations. For a forum on adolescent HIV, there were no participants aged 10-19 years: <10% were 20-24 years old, and less than a third of participants were female. Adolescent girls and young women carry a disproportionally high burden of HIV in African countries and LMICs and bear a significant responsibility in preventing perinatal HIV transmission [2]; they were, unfortunately, under-represented in this forum. However, overall, nearly half of our participants were aged <35 years. Furthermore, we assigned youth moderators and participants to each group to amplify the voices of AY participants. Despite these limitations, this stakeholder consultation adds early, youth-focused, multicountry perspectives to the growing body of information on the implementation of LA ART in LMICs.

## Conclusion

LA ART presents an attractive alternative to daily oral treatment for HIV, especially for adolescents and youth with adherence issues. Our stakeholder consensus findings come at a time when most LMICs, including high-burden African countries, are still at the pre- or early implementation stage of LA ART for any population; even high-income countries with LA ART approval are reporting implementation challenges with logistics and uptake [47]. Guided by lessons learned from consultations such as we have described, the implementation of LA ART for AY will allow HIV programs to enhance community engagement, strengthen AY-focused health services, negotiate affordable costs, and invest in quality and sustainable logistics and supply chains. We now have a window of opportunity for early and meaningful engagement with AY and other key stakeholders to achieve equitable LA ART implementation for current and future generations.
